# Design of High-Precision Driving Control System for Charge Management

**DOI:** 10.3390/s24092883

**Published:** 2024-04-30

**Authors:** Yang Wang, Boyan Lv, Tao Yu, Longqi Wang, Zhi Wang

**Affiliations:** 1 College of Engineering and Technology, Jilin Agricultural University, Changchun 130118, China; wangyang800507@126.com (Y.W.);; 2 Changchun Institute of Optics, Fine Mechanics and Physics, Chinese Academy of Sciences, Changchun 130033, China; 3 School of Fundamental Physics and Mathematical Sciences, Hangzhou Institute for Advanced Study, UCAS, Hangzhou 310024, China

**Keywords:** charge management, UV-LED constant current source, PWM

## Abstract

Due to the interaction of accumulated charges on the surface of a test mass with the surrounding electric and magnetic fields, the performance of inertial sensors is affected, necessitating charge management for the test mass. Discharge technology based on Ultraviolet LEDs is internationally recognized as the optimal solution for charge management. Precision driving of Ultraviolet LEDs is considered a key technology in charge management. This paper presents the driving control system used for Ultraviolet LEDs, achieving precision pulse-width-modulation-type current output with controllable pulse width and amplitude. The system generates the pulse-width-controllable pulse voltage signal via analog pulse-width modulation, and subsequently regulates the amplitude of the PWM signal through range switching. To convert the voltage into the pulse-width-modulation-type driving current, the improved Howland current source is employed. The test results demonstrate that the driving control system can output controllable current in the range of 0.01 mA to 10 mA, with a minimum step of 0.01 mA. The accuracy of the current reaches 1%, the stability within 1 h is better than 1%, and the load regulation is better than 2%. The driving control system provides an important reference for the integration of charge management system and the precision drive control method for LEDs.

## 1. Introduction

The inertial sensor is a core component of the space gravitational wave detection mission known as “Taiji” [1,2]. The test mass (TM) within the inertial sensor serves as the inertial reference for space gravitational wave detection. However, the surface of the TM is susceptible to the accumulation of charges from high-energy solar particles, cosmic rays, and other sources [3]. These accumulated charges couple with residual Direct Current (DC) electric fields on nearby surfaces and interplanetary magnetic fields, exerting forces on the TM. This interaction leads to the generation of acceleration noise on the TM, thereby interfering with gravitational wave detection [4,5]. Therefore, it is necessary to perform “charge management” on the surface of the TM, specifically implementing control over its charging and discharging, to mitigate the impact of charges on scientific detection tasks [6]. The method based on the photoelectric effect principle, utilizing ultraviolet light sources to irradiate the TM and its external electrode housing, is considered an effective solution [7,8,9]. The implementation of this method involves no mechanical connections between the charge management system (CMS) and the TM, ensuring that charge management is achieved without introducing additional displacement noise interference [10,11]. Currently, Ultraviolet LEDs (UV-LEDs) are chosen as ultraviolet light sources due to their high reliability, low power consumption, long lifespan, and resistance to radiation—making them highly suitable for space exploration tasks [12]. The use of UV-LEDs as the light source has been validated in other space missions [13,14]. In the CMS, UV-LEDs need to output precise and adjustable light power to control the charge on the TM surface. Since the light power from the UV-LED is directly proportional to its driving current, the driving control system is required to provide stable, accurate, and adjustable output current to the UV-LED [15].

Regarding the UV-LED driving control strategy for the CMS, Ke et al. proposed strategies for rapid DC discharge and continuous pulse discharge for CMS in the “ Laser interferometer space antenna (LISA)” [16]. Continuous pulse discharge poses a challenge for the implementation of driving control in CMS. Samantha et al. focused on continuous pulse discharge, proposing the continuous pulse discharge method using pulse width modulation (PWM) for UV-LED control. A Technical Readiness Level (TRL) 4 experimental prototype for the CMS of LISA was designed and built by them. The UV-LED current amplitude was regulated by controlling the output DC current, and the UV-LED pulse current width was further controlled by adjusting the frequency of the parallel switch connected to the UV-LED. A maximum output frequency of 100 KHz, a maximum current amplitude of 50 mA, and a step of 12.2 µA were achieved [17]. The dimming method of the TRL4 prototype was further optimized by them, and the TRL5 prototype was designed and constructed, achieving a constant current system with performance similar to the TRL4. The advantages of the TRL5 prototype lie in its capability to directly output the PWM current with adjustable pulse amplitude and pulse width, thereby enhancing system stability and reliability while further reducing power consumption. This improvement enhances system stability, reliability, and further reduces power consumption. Reference [18] used the TRL5 driving control system to test UV-LEDs to compare the performance of UV-LEDs under different driving currents. The individual LED driving current ranged from 0 to 12.5 mA. The test results revealed significant differences in the spectrum, light power, and pulse characteristics of LEDs from the same batch at low driving currents. Yang Q developed a prototype UV light source system to enhance the fiber coupling efficiency of the “Tian Qin “ [19] CMS [20]. The system generated PWM voltage signals using a signal generator, which were then converted by the driving control system into a pulse current with the frequency of 1 KHz, amplitude of 1 mA, and adjustable duty cycle (pulse width) from 0% to 100%. The results indicated that this light source system met the functional requirements for verifying fiber coupling efficiency. Tian J proposed a pulse discharge control method for a charge management technology verification satellite, creating an experimental prototype. Using an STM32 to control the UV-LED driving chip, they achieved a pulse current with the frequency of 1 KHz, the amplitude of 20 mA, and the adjustable duty cycle from 0% to 100%. Experimental results demonstrated that the prototype realized power control of UV-LEDs under different duty cycles, with higher duty cycles leading to faster charging and discharging rates [21].

In summary, the continuous pulse discharge strategy of the CMS adopts PWM-type current output, with the output current amplitude not exceeding 50 mA. These LED driving control methods are more focused on achieving signal transformation functions, most of which are designed with open-loop circuits. This paper introduces a closed-loop control method based on signal transformation to improve the stability of the driving control system, which is more conducive to practical applications. A continuous pulse driving control method for CMS is proposed in this paper. This driving control method not only enables precise dimming of CMS but also provides a high-precision implementation method for LED driving control, which can be extended to industrial light curing, crop supplementary lighting, UV sterilization, and other fields [22,23,24].

## 2. Design of Charge Management Driving Control System

To verify the feasibility of the proposed driving control method, the pulse frequency of the driving control system was set to 1 KHz. Since the system serves one LED, the output voltage of the driving control system does not need to be excessively high. To minimize the impact of the pulse control method on the LED’s lifespan, the pulse amplitude was kept as close to the rated LED current as possible. Additionally, for precise control of the output current, the current fluctuation should be minimized. Considering the current functional verification requirements, the driving control system is required to output a PWM-type pulse current at 1 kHz, with adjustable amplitude and width. The average output current range was set between 0 and 10 mA, with a minimum step of 0.01 mA. Therefore, the output current fluctuation should be less than 0.01 mA, the output accuracy should reach 1%, and the output stability within the 1-h range should achieve 1%. As the LED serves as the load for the driving control system, operating at the frequency of 1 kHz, the LED load can be equivalently modeled as a dynamic resistor during design. Hence, the system has load regulation requirements to meet the specifications of a typical constant current source, with the load regulation being less than 5%. In the selection of the design scheme, traditional PWM-type LED dimming systems often use buck/boost converters as the basis for outputting driving current. They connect LEDs with switching transistors in series or parallel to achieve PWM dimming. However, this dimming method has been proven to require additional power consumption, have potential instability, and is commonly used in situations involving high currents or multiple LEDs [17]. Directly adopting high-precision constant current sources capable of outputting PWM waveforms is costly, bulky, and has limited output frequencies, making it less suitable for subsequent high-frequency applications and CMS integration.

**Figure 1:**
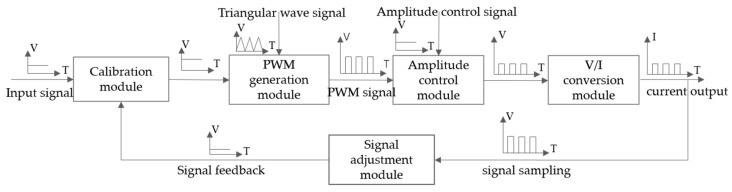
Driving control system for CMS.

Therefore, a dedicated driving control system is designed to achieve high integration, high precision, and low cost. Compared to traditional LED driving methods primarily based on switch converters, this driving control system eliminates dimming switch transistors that may cause disturbances. Inspired by the constant current drive method, and combining the closed-loop strategy of the converter with the current range conversion function of the constant current source, a high-precision, wide-range, PWM-type driving control system for CMS was designed. The schematic of the driving control system is shown in Figure 1. Adjusting the system’s input voltage signal changes the PWM pulse current duty cycle (pulse width), and adjusting the amplitude control signal changes the PWM pulse current amplitude.

**Figure 2:**
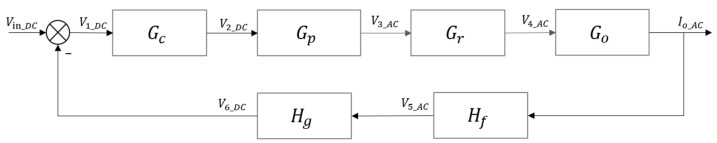
Structure diagram of the driving control system.

The calibration module utilizes the proportional controller to correct the input signal, and the PWM generation module generates PWM signals based on analog pulse width modulation. The amplitude control module adjusts the amplitude of the PWM signal using the resistor divider network. The V/I conversion module achieves voltage-to-current conversion based on the Howland current source. The signal adjustment module is primarily used to convert the sampled PWM signal into an equivalent DC signal for closed-loop control. Based on the aforementioned driving control structure, the system diagram in Figure 2 is obtained.

**Figure 3:**
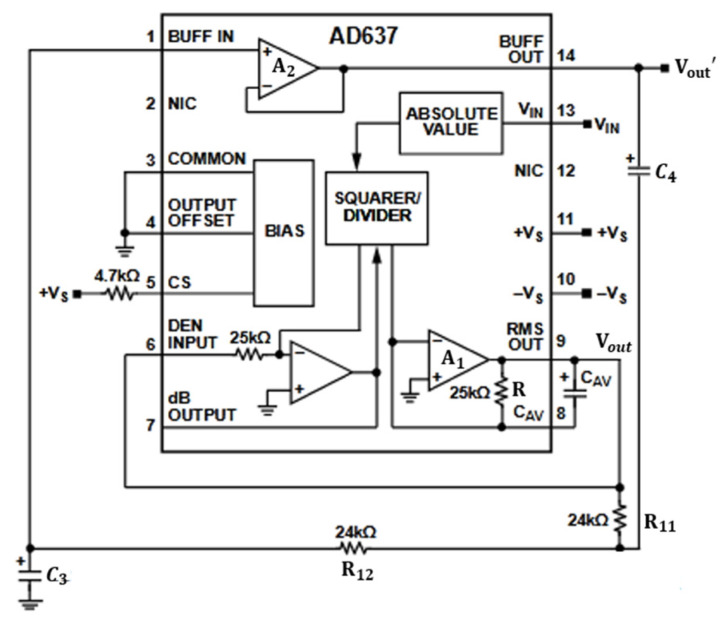
Effective value circuit with second-order Sallen–Key filtering.

The transfer functions for the calibration module and amplitude adjustment module are given by $G_{c}=\frac{V_{2}}{V_{1}}=K_{1}$ and $G_{R}=\frac{V_{4}}{V_{3}}=K_{2}$, respectively. The PWM generation module has the transfer function $G_{p}=\frac{V_{3}}{V_{2}}=-\frac{R_{2}}{R_{1}}$. Since the feedback loop requires the sampling resistor $R_{3}$, the transfer function for the feedback loop is $H_{f}=\frac{V_{5}}{I_{o}}=R_{3}$. The V/I conversion module has the transfer function $G_{o}=\frac{I_{o}}{V_{4}}=\frac{1}{R_{3}}$. The signal adjustment module, denoted as $H_{g}$, is an effective value circuit implemented using the AD637 and its peripheral circuitry [25], as shown in Figure 3.

AD637 is the true Root Mean Square (RMS) circuit, meaning it performs the Root Mean Square operation and can transform an AC signal into its DC effective value signal. According to the datasheet, AD637 employs the circuit with low-pass filtering to achieve the averaging operation. Specifically, when $C_{AV}$ is sufficiently large, its output represents the average DC voltage. To further improve the quality of the output signal, it internally integrates an operational amplifier $A_{2}$ that, together with external resistors and capacitors, forms a second-order Sallen–Key filter.

Analyzing the working principle of this stage reveals the input–output relationship, as follows:

(1) $$RC_{AV}V_{out}\left(t\right)\frac{{dV}_{out}\left(t\right)}{dt}+{\left(V_{out}\left(t\right)\right)}^{2}={\left(V_{in}\left(t\right)\right)}^{2}.$$

According to small-signal modeling [26], processing Formula (1) and solving it using the Laplace transform results in the following equation:

(2) $$\left(sRC_{AV}U_{out}+2U_{out}\right){\hat{V}}_{out}\left(s\right)=2U_{in}{\hat{V}}_{in}\left(s\right)$$

where $U_{in}$ is the effective value of the input AC signal and $U_{out}$ is the DC effective value output. Therefore,

(3) $$\frac{{\hat{V}}_{out}\left(s\right)}{{\hat{V}}_{in}\left(s\right)}=\frac{1}{\frac{RC_{AV}}{2}s+1}.$$

With $A_{2}$ enabled, the SK type second-order, low-pass filter is connected in series after the effective value operation. Therefore, the transfer function of the signal adjustment section is

(4) $$H_{g}=\frac{1}{\left(\frac{RC_{AV}}{2}s+1\right)*\left(R_{11}R_{12}C_{3}C_{4}s^{2}+R_{11}R_{12}C_{3}C_{4}s+1\right)}.$$

According to the analysis of the above driving control system block diagram, the input–output transfer function of the system is

(5) $$\frac{I_{out}}{V_{in}}=\frac{G_{c}G_{p}G_{r}G_{o}}{1-G_{c}G_{p}G_{r}G_{o}H_{f}H_{g}}\;=\frac{K_{1}K_{2}RR_{11}R_{12}{R_{2}C}_{AV}C_{3}C_{4}s^{3}+K_{1}K_{2}R_{11}R_{12}R_{2}C_{3}C_{4}\left(RC_{AV}+2\right)s^{2}+K_{1}K_{2}R{R_{11}R_{12}R_{2}C}_{AV}C_{3}C_{4}s+2K_{1}K_{2}R_{2}}{RR_{1}R_{11}R_{12}R_{3}C_{AV}C_{3}C_{4}s^{3}+R_{1}R_{11}R_{12}{R_{3}C}_{3}C_{4}\left(RC_{AV}+2\right)s^{2}+RR_{1}{R_{11}R_{12}R}_{3}C_{AV}C_{3}C_{4}s+2R_{1}R_{3}(1+K_{1}K_{2}R_{2})}.$$

To achieve PWM-type output current, the core components of the driving control system are the PWM generation module and the V/I conversion module. The following provides an introduction to these two modules.

### 2.1. PWM Generation Module

**Figure 4:**
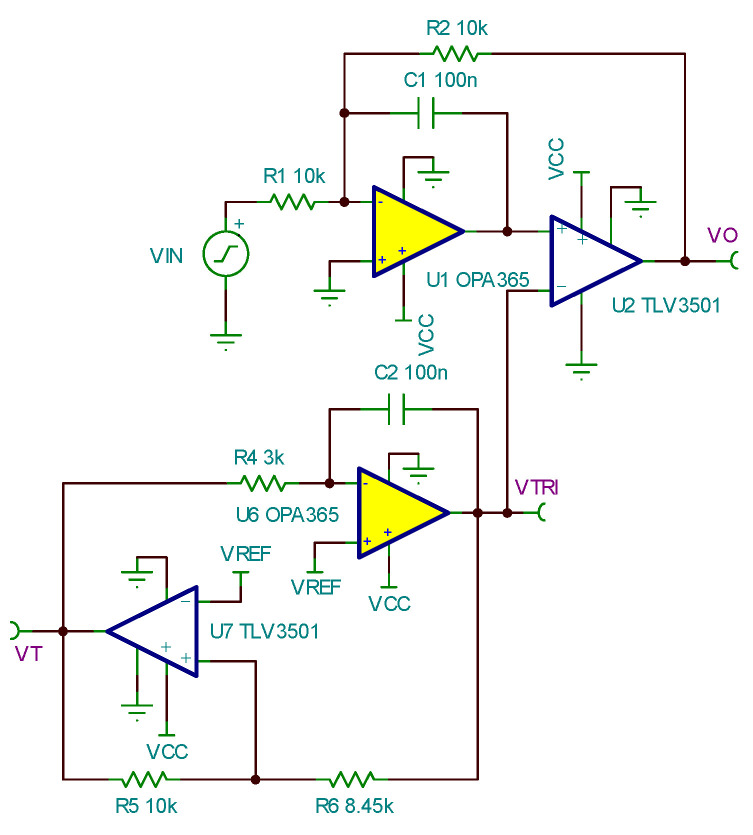
PWM generation circuit.

The PWM generation module employs the analog PWM circuit, and its implementation circuit is shown in Figure 4. This circuit can achieve PWM output with an amplitude of 5 V, a frequency of 1 KHz, and a duty cycle ranging from 0 to 100%. Operational amplifier U6 and comparator U7 are used to generate the triangular wave, which is compared with the input signal at the positive input terminal of U2 to generate the PWM signal. U1 serves as the error amplifier, providing negative feedback to the PWM circuit to ensure high precision and linearity in the output. The input–output relationship of the PWM generation circuit is as follows:

(6) $$V_{o}=-\frac{R_{2}}{R_{1}}\cdot V_{in}.$$

In the equation, $V_{in}$ represents the input DC signal and $V_{o}$ represents the output average voltage signal of PWM.

According to the principles of PWM,

(7) $$V_{o}=\delta \cdot V_{T}.$$

In the equation, δ represents the duty cycle of the output PWM signal and $V_{T}$ represents the amplitude of the output PWM signal.

Substituting Formula (7) into (6), we obtain

(8) $$\delta =-\frac{R_{2}}{R_{1}V_{T}}\cdot V_{in}.$$

In this design, the amplitude of the output PWM signal $V_{T}$ is fixed at 5 V. Therefore, from Formulas (6) and (8), it is evident that $V_{in}$ controls the average PWM output voltage, influencing the duty cycle, while the output PWM frequency is determined by the triangular wave generator. The following provides a detailed introduction to the implementation circuit.

Operational amplifier $U_{1}$, together with external resistors and capacitors, forms the bandwidth-limited inverting amplification circuit. The PWM signal output from $U_{2}$ is filtered through $R_{2}$ and $C_{1}$ to convert it into the average value, which is then fed back to the input to implement closed-loop control, enhancing system accuracy. The break frequency f is determined according to Formula (9):

(9) $$f=\frac{1}{2\pi {C_{1}R}_{2}}.$$

Among them, the selection range of f should be far below the 1 KHz output frequency. To achieve waveform transformation, the capacitance $C_{1}$ is chosen to be 100 nF.

The PWM signal frequency is determined using the triangular wave generator circuit composed of $U_{6}$, $U_{7}$, and their external resistors and capacitors. Comparator $U_{7}$, together with $R_{5}$ and $R_{6}$, forms an in-phase input hysteresis comparator. Since the circuit is powered by the single 5 V supply and to achieve the triangular wave output with an amplitude of 0~5 V, the 2.5 V voltage reference $V_{ref}$ is added. Therefore,

(10) $$V_{TRI}=\frac{R_{6}}{R_{5}}V_{ref}.$$

In the equation, $V_{TRI}$ represents the amplitude of the triangular wave output.

The operational amplifier $U_{6}$, together with $R_{4}$ and $C_{2}$, forms the integrator circuit. Therefore, the frequency of the triangular wave generator is

(11) $$f_{T}=\frac{R_{5}}{4R_{4}C_{2}R_{6}}.$$

### 2.2. V/I Conversion Circuit

**Figure 5:**
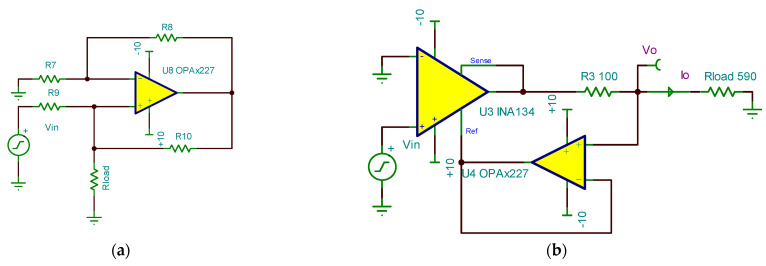
(**a**) Howland current source. (**b**) Improved Howland current source.

The V/I conversion circuit in this system uses the Howland current source. The Howland current source is illustrated in Figure 5a. When the condition $R_{7}/R_{9}=R_{8}/R_{10}$ is met, the current on the load resistor $R_{load}$ is given by $I_{load}=U_{in}/R_{9}$. However, the Howland circuit requires a high degree of matching for the resistors $R_{7}$ to $R_{10}$. Tiny differences in these resistors can lead to inaccurate outputs, making implementation challenging. Figure 5b depicts the improved Howland current source, utilizing the integrated combination of differential amplifiers and voltage followers. This enhances the feasibility of the Howland current source implementation. This modification enhances the feasibility of the Howland current source.

$U_{5}$ is the differential operational amplifier with four laser-trimmed 25 kΩ precision resistors and is used to address the resistor matching issue in the Howland current source. $U_{6}$ forms the voltage follower. Therefore, the load current for the V/I conversion module is

(12) $$I_{load}=\frac{V_{in}}{R_{3}}.$$

In this equation, $V_{in}$ is the input signal from the preceding module.

At this point, the load current is only related to the input voltage $V_{in}$ and the sampling resistor $R_{3}$, independent of the size of the load resistor. The four resistor matching issues in the Howland circuit are simplified to the selection of $R_{3}$. Because $V_{in}$ is the PWM signal output from the preceding stage, and the accuracy of $R_{3}$ affects the accuracy of the output current, the high-precision, non-wire wound resistor should be chosen for $R_{3}$. Considering the output current requirements, the 0.05% precision, 100 Ω metal film resistor is selected as the sampling resistor.

**Figure 6:**
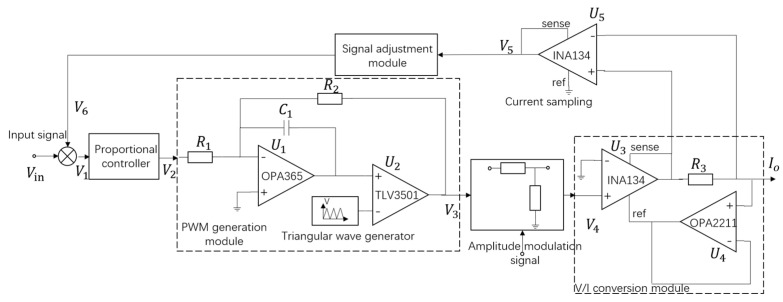
Block diagram of charge management driving control system.

According to the above analysis, the system block diagram is obtained as shown in Figure 6:

## 3. Circuit Parameter Setting and Simulation

According to the theoretical model of the system and the control system diagram, simulations were conducted in TINA-TI. Based on the requirements outlined in Section 1, the output should range from 0.01 mA to 10 mA average current, with adjustable PWM duty cycle and amplitude. Inspired by the range conversion function of the constant current source control method and to verify its feasibility, PWM output current waveforms were simulated for two amplitude levels: 1 mA and 10 mA. Each amplitude level can achieve adjustable duty cycles from 0% to 100%, with a step size of 1%. Therefore, this control system can output average currents in two ranges. When the output amplitude is set to 1 mA, the average output current ranges from 0.01 mA to 1 mA, with a step size of 0.01 mA. When the output amplitude is set to 10 mA, the average output current ranges from 0.1 mA to 10 mA, with a step size of 0.1 mA. In the driving control system, due to the characteristics of comparator $U_{2}$ output, the PWM generation module continuously outputs the PWM-type voltage signal with an amplitude of 5 V. According to Equation (6), adjusting the input signal from −5 V to 0 V results in the average output voltage signal varying from 0 V to 5 V. The V/I conversion module has two input amplitudes, 0.1 V and 1 V, and, according to Equation (7), the output current signal amplitudes are 1 mA and 10 mA, respectively.

However, the simulation model for AD637 does not support simulations other than sinusoidal signals. Analyzing Equation (12), it was found that the low-pass filter can be used as a substitute. Based on the overall scheme of the control system and the parameter settings mentioned above, the simulation was conducted.

**Figure 7:**
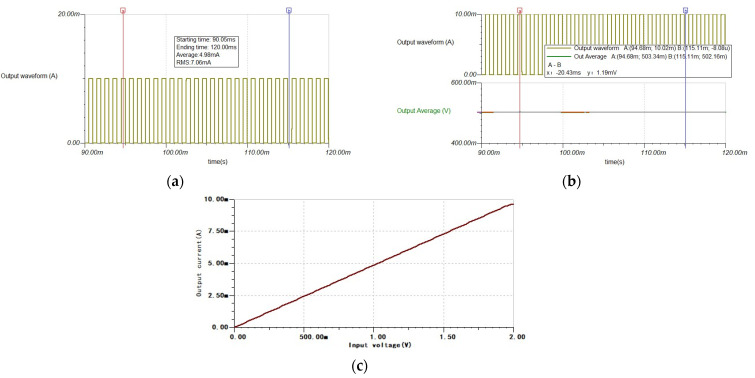
(**a**) The output pulse current waveform has an amplitude range of 10 mA with a duty cycle of 50%. (**b**) Output pulse current amplitude. (**c**) Input–output relationship at the 10 mA range.

**Figure 8:**
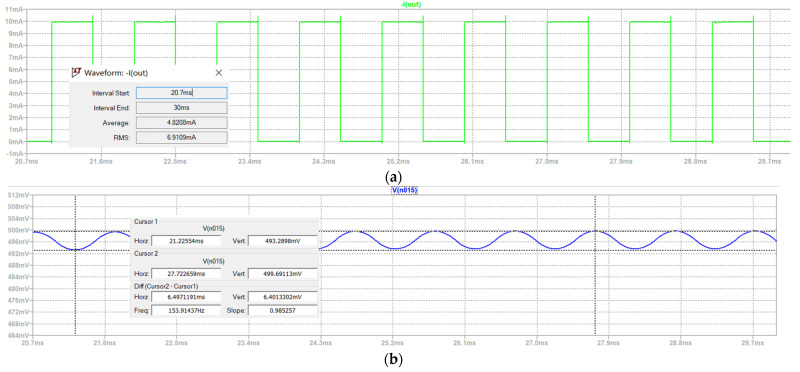
(**a**) The waveform of the output current in the traditional method. (**b**) Output pulse current amplitude.

The simulation results of the output, with an amplitude of 10 mA and a 50% duty cycle, are shown in Figure 7, as an example. We adopted the traditional method of serially connecting switching tubes based on switch converters to achieve pulse current output, while meeting the requirements of charge management functions; the simulation output results are shown in Figure 8. For the convenience of accuracy and stability evaluation, the output current was processed according to the reference standard “JJG70-2015 < Regulations for the Calibration of AC Standard Current Sources>”, with specific details provided in Section 4.1. For observing the stability of the average output current, both output currents are amplified by 100 times and processed using a fourth-order, MFB-type, low-pass filter with a cutoff frequency of 100 Hz.

From the simulation results, it can be seen that, after the stability of the two systems’ outputs, the average value of the output pulse current, shown in Figure 7a, is 4.98 mA with an accuracy of 0.40%; in Figure 8a, it is 4.82 mA with an accuracy of 3.7%. According to the standard, the maximum and minimum points of the output after stability are selected for testing. The output fluctuation shown in Figure 7b is 1.19 mV, with an actual output fluctuation of 11.9 uA and a stability of 0.24%. In Figure 8b, the output fluctuation is 6.40 mV, with an actual output fluctuation of 64 uA and stability of 1.3%. Therefore, the accuracy of the average output current of the driving control system is better than that of the traditional method, while the amplitude fluctuation of the output of the driving control system is significantly better than that of the traditional method. Therefore, adopting the driving control scheme can meet the expected output requirements and is more suitable for high-precision pulse current output applications.

Taking 10 mA amplitude output as an example, the relationship between different input voltages and the average output current is plotted as shown in curve 7(b). The linear relationship between input and output lines indicates that the expected current output can be easily achieved by adjusting the input voltage.

## 4. Experimental Results and Analysis

**Figure 9:**
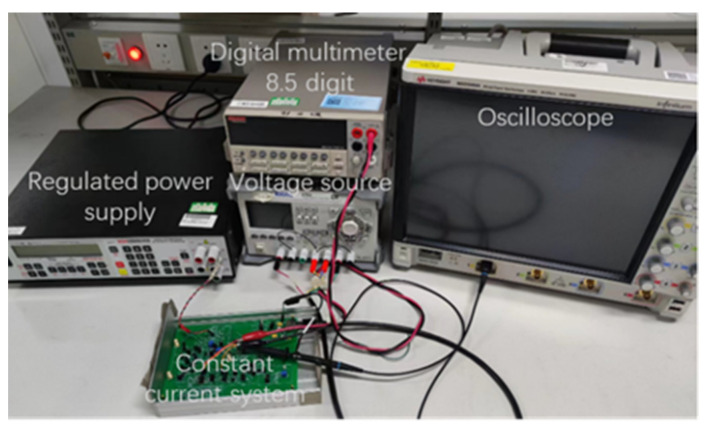
Experimental testing environment.

To validate the proposed driving control method after simulation analysis, the prototype of the driving control system was designed and constructed, and the experimental testing system was set up, as shown in Figure 9. The input signal was provided via Krohn–Hite’s Model 523 voltage source meter, the output waveform was observed using Keysight’s MSOS404A oscilloscope, and the output current was monitored using Keithley’s 8.5 digit Model 2002 High Performance Digital multimeter. The driving control system was powered using Rigol’s DP832 regulated power supply. To reduce interference from electromagnetic environments and airflow disturbances in the laboratory, the driving control system was placed in a dedicated aluminum shielding box. Since the output current of the driving control system was relatively weak, an indirect method was used to measure the output current. The PWM sampling circuit was reused, converting the output current to the voltage value through a 100 Ω resistor. The MOS404A oscilloscope was connected to the output of the sampling circuit to observe the output waveform. The 2002 multimeter was connected to the output of the signal adjustment module to accurately measure the magnitude of the output current.

### 4.1. Evaluation Method for Test Results

This design can output stable current with a PWM waveform. Its evaluation is based on the national standard “JJG70-2015 <Calibration Procedures for AC Standard Current Sources>”. Combining the analysis results from Section 1, the driving control system was subjected to tests for the resolution, the accuracy, the stability, and the load regulation to verify if it meets the requirements. The calculation methods for the accuracy, the stability, and the load regulation are as follows:

The accuracy:

(13) $$\gamma =\frac{\left|I_{t}-{\bar{I}}_{r}\right|}{{\bar{I}}_{r}}\times 100\%.$$

In the equation, $I_{t}$ is the reference value of the output current and ${\bar{I}}_{r}$ is the average measured value of the output current, where

$${\bar{I}}_{r}=\frac{1}{n}\sum_{n}I_{r}.$$

In the equation, n represents the actual number of measurements. In this experiment, within the 1-h range, n≈900.

The stability:

(14) $$S=\frac{I_{MAX}-I_{MIN}}{{\bar{I}}_{r}}\times 100\%.$$

In the equation, $I_{MAX}$ and $I_{MIN}$ are the maximum and minimum values of the output current within 1 h, respectively.

The load regulation:

(15) $$LR=\frac{{\bar{U}}_{F}-{\bar{U}}_{E}}{{\bar{U}}_{E}}\times 100\%.$$

In the equation, ${\bar{U}}_{F}$ represents the average measured output voltage when the load resistor is at its rated value, and ${\bar{U}}_{E}$ represents the average measured output voltage when the load resistor is short-circuited, where

$${\bar{U}}_{F}=\frac{1}{n}\sum_{n}U_{F},{\bar{U}}_{E}=\frac{1}{n}\sum_{n}U_{E}.$$

In the equation, $U_{F}$ is the output voltage when the load resistance is at its rated value; $U_{E}$ is the output voltage when the load resistance is short-circuited.

Extracting nine different duty cycle outputs—1%, 10%, 20%, 30%, 40%, 50%, 70%, 90%, 98% of continuous data collection for 1 h for each output—the accuracy, the stability, and the load adjustment rate are calculated based on the established test environment and evaluation method mentioned above.

### 4.2. Analysis of Test Results

**Figure 10:**
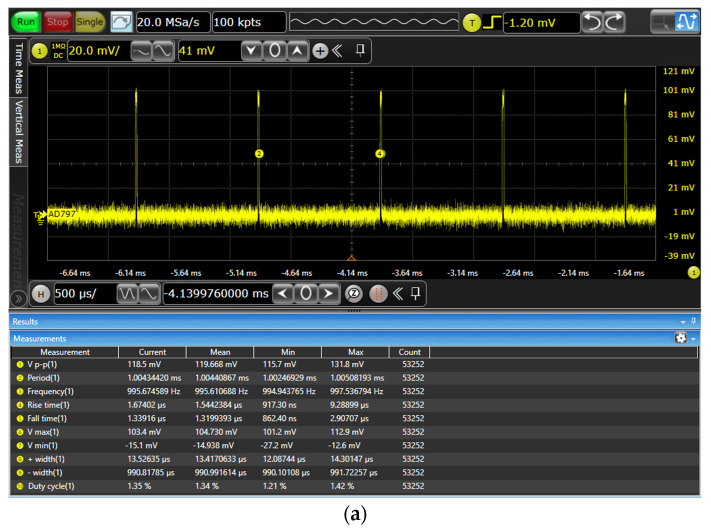

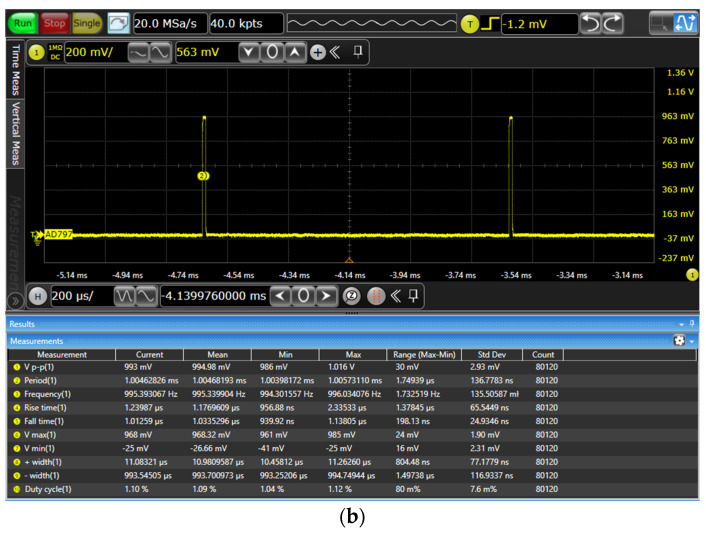
(**a**) The 1% duty cycle 1 mA amplitude output waveform. (**b**) The 1% duty cycle 10 mA amplitude output waveform.

Taking the example of the 1% duty cycle with 1 mA and 10 mA amplitude outputs, continuous 1-h testing was conducted on the driving control system. The oscilloscope’s output results are shown in Figure 10. The theoretical values were 0.01 mA and 0.1 mA, and the actual average output currents were 0.009694818 mA and 0.097749533 mA, demonstrating that the output duty cycle resolution can achieve 1%.

Accurate testing was conducted based on the content outlined in Section 4.1, and the experimental data are presented in Table 1:

Accurate testing was conducted based on the content outlined in Section 4.1, and the experimental data are presented in Table 1: these data represents the continuous 1-h testing of nine different output duty cycles selected under 1 mA and 10 mA output amplitudes. The continuous data output was averaged to obtain the measured result for each duty cycle. Following the calculation method in Section 4.1, the output accuracy reaches 1%. Additionally, as previously analyzed, the stability is a more critical metric for testing this driving control system. The output stability is presented in Table 2.

Table 2 was conducted concurrently with the accuracy test. Under conditions where there is no temperature control system to maintain a constant temperature in the testing environment, the stability of the driving control system is less than 1%, meeting the expected target requirements. The output load regulation is presented in Table 3.

After conducting the accuracy and stability tests, the current load output conditions were recorded. Subsequently, the equivalent load was short-circuited, and output data collection was performed. Based on the characteristics of the LED to be driven, it is known that, under the conditions of 10 mA output, the equivalent load is approximately 590 Ω; under the conditions of 1 mA output, the equivalent load is approximately 1 KΩ. Therefore, these two resistor values were selected as loads for testing, and the results show that the load adjustment rate is less than 2%, meeting the typical load adjustment rate requirements for constant current sources.

**Figure 11:**
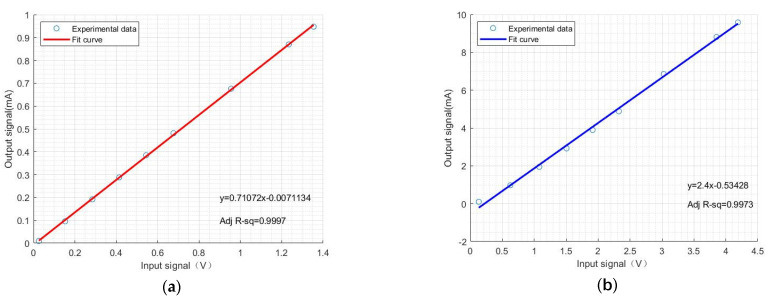
(**a**) The input–output relationship of actual measurement in the 1 mA range. (**b**) The input–output relationship of actual measurement in the 10 mA range.

From the previous simulation analysis, it is evident that there is a linear relationship between the input and output of the driving control system. Therefore, the collected data are fitted using the least squares method and the results are shown in Figure 11.

From Figure 11, it can be observed that the input–output relationship exhibits linear variation, and the regression line is given by

(16) $$I_{o}\left(x_{i}\right)=A*x_{i}-B.$$

In the equation, $I_{o}\left(x_{i}\right)$ represents the average output current, $x_{i}$ represents the input voltage and A and B are the coefficients of the fitted line. For the 1 mA range, A = 0.7107 and B = 0.007113; for the 10 mA range, A = 2.400 and B = 0.5343. And, the R-Square of the two curves is 0.9997 and 0.9973, respectively. This indicates that the desired average output current can be obtained according to Formula (16).

Comparing the output data between the 1 mA and 10 mA ranges, it is evident that the accuracy and the stability test results for the 10 mA range are significantly better than those for the 1 mA range. The reason for this is that the output current is weaker at the 1 mA range, making it more susceptible to external environmental interference. While good grounding methods and electromagnetic shielding measures have been implemented to reduce most electromagnetic interference, factors such as temperature variations in the testing environment and minor vibrations in the testing platform may still affect the driving control system. There are more data in the Supplementary File.

## 5. Conclusions

The driving control system plays a pivotal role in charge management, facilitating stable and accurate control of the current supplied to UV-LEDs. This enables contactless charge control of the TM. The proposed method, centered around the constant current implementation with the PWM generation circuit and the improved Howland circuit, addresses the challenge of achieving high-precision PWM current with adjustable pulse width and pulse amplitude.

The experimental validation of this approach encompassed assessments of the output accuracy, the stability, the resolution, and the load regulation. The results demonstrate the system’s capability to output a stable and accurate PWM-type current within the average current range of 0.01 mA to 10 mA. In the absence of temperature control, the output current accuracy achieved 1%, surpassing the stability requirement of 1% and meeting the specified performance criteria. The load regulation was found to be less than 2%, aligning with standard current source specifications. The observed linear input–output relationship curve facilitates programmable control and accurate stepping.

This study has implemented a drive control solution tailored for the CMS, and the proposed driving control system extends its applicability to other scenarios requiring precise control of pulsed current. Potential applications include plant supplemental lighting, UV sterilization, and disinfection processes.

## Figures and Tables

**Table 1:** The output Accuracy.

Output Amplitude/mA	Output Duty Cycle/%	$I_{t}$/mA	${\bar{I}}_{r}$/mA	γ/%
0.96850931				
	1	0.009685093	0.009694818	0.10031029
	10	0.096850931	0.095626182	1.280767124
	20	0.193701862	0.191838931	0.971091317
	30	0.290552793	0.288182208	0.822599356
	40	0.387403724	0.384969933	0.632202793
	50	0.484254655	0.481217581	0.631122753
	70	0.677956517	0.675934623	0.299125606
	90	0.871658379	0.869889733	0.203318394
	98	0.949139124	0.947377765	0.185919369
9.77829041				
	1	0.097782904	0.097749533	0.034139396
	10	0.977829041	0.971700619	0.630690341
	20	1.955658082	1.946204522	0.485743383
	30	2.933487123	2.92406579	0.32219975
	40	3.911316164	3.897138722	0.363791046
	50	4.889145205	4.88333694	0.118940495
	70	6.844803287	6.851809943	0.10225993
	90	8.800461369	8.808456174	0.09076284
	98	9.582724602	9.580863813	0.019421928

**Table 2:** The output stability.

Output Amplitude/mA	Output Duty Cycle/%	${\bar{I}}_{r}$/mA	$I_{MAX}$/mA	$I_{MIN}$/mA	S/%
0.96850931					
	1	0.009694818	0.009707689	0.009681364	0.271536815
	10	0.095626182	0.09573407	0.095525533	0.218074585
	20	0.191838931	0.192046603	0.191575128	0.245766035
	30	0.288182208	0.288546958	0.287876393	0.232688124
	40	0.384969933	0.38530072	0.38454381	0.196615389
	50	0.481217581	0.48208972	0.480490619	0.332303065
	70	0.675934623	0.677087156	0.675007002	0.307744904
	90	0.869889733	0.873055812	0.867560754	0.631695956
	98	0.947377765	0.949918124	0.942078348	0.827523783
9.77829041					
	1	0.097749533	0.097816944	0.097683847	0.136161264
	10	0.971700619	0.972433569	0.970909563	0.156839044
	20	1.946204522	1.94721895	1.94538715	0.094121675
	30	2.92406579	2.926158907	2.92187369	0.146549951
	40	3.897138722	3.899581942	3.892150672	0.19068529
	50	4.88333694	4.887003791	4.875768705	0.230069836
	70	6.851809943	6.861267431	6.844152454	0.249787677
	90	8.808456174	8.822764918	8.795845659	0.305606999
	98	9.580863813	9.609864829	9.535931421	0.771677891

**Table 3:** The output load regulation.

Output Amplitude/mA	Output Duty Cycle/%	${\bar{I}}_{r}$/mA	${\bar{U}}_{F}$/mA	${\bar{U}}_{E}$/mA	LR/%
0.96850931					
	1	0.009694818	0.009694011	0.009879509	1.877603432
	10	0.095626182	0.095334481	0.095572319	0.248856643
	20	0.191838931	0.191701109	0.192025423	0.168891609
	30	0.288182208	0.287666483	0.28784704	0.062726628
	40	0.384969933	0.383816366	0.384138178	0.083775153
	50	0.481217581	0.480990388	0.481250384	0.054022512
	70	0.675934623	0.675347054	0.675541611	0.028800139
	90	0.869889733	0.868953933	0.868607626	0.039869251
	98	0.947377765	0.947052517	0.948318059	0.133451169
9.77829041					
	1	0.097749533	0.09859061	0.099463174	0.877273432
	10	0.971700619	0.975912801	0.977671692	0.179906043
	20	1.946204522	1.952617368	1.955250316	0.134660389
	30	2.92406579	2.925964492	2.928855202	0.098697602
	40	3.897138722	3.904120949	3.907301694	0.081405164
	50	4.88333694	4.882806511	4.886662286	0.078904071
	70	6.851809943	6.839367345	6.844018555	0.067960228
	90	8.808456174	8.75862369	8.777457065	0.214565273
	98	9.580863813	9.602421646	9.586678173	0.164222407

## Data Availability

The original contributions presented in the study are included in the article, further inquiries can be directed to the corresponding author.

## Supplementary Materials

The following supporting information can be downloaded at: https://www.mdpi.com/article/10.3390/s24092883/s1, Supplementary File is about experimental data.

## Abbreviations

The following abbreviations are used in this manuscript:

CMS	Charge management system
DC	Direct Current
LISA	Laser interferometer space antenna
PWM	Pulse width modulation
TM	Test mass
TRL	Technical Readiness Level
RMS	Root Mean Square
UV	Ultraviolet
