# Supplementary figures and images for: Design of High-Precision Driving Control System for Charge Management
Source: Sensors (Basel). 2024 Apr 30;24(9):2883. doi: 10.3390/s24092883 (PMC11086223; doi:10.3390/s24092883)

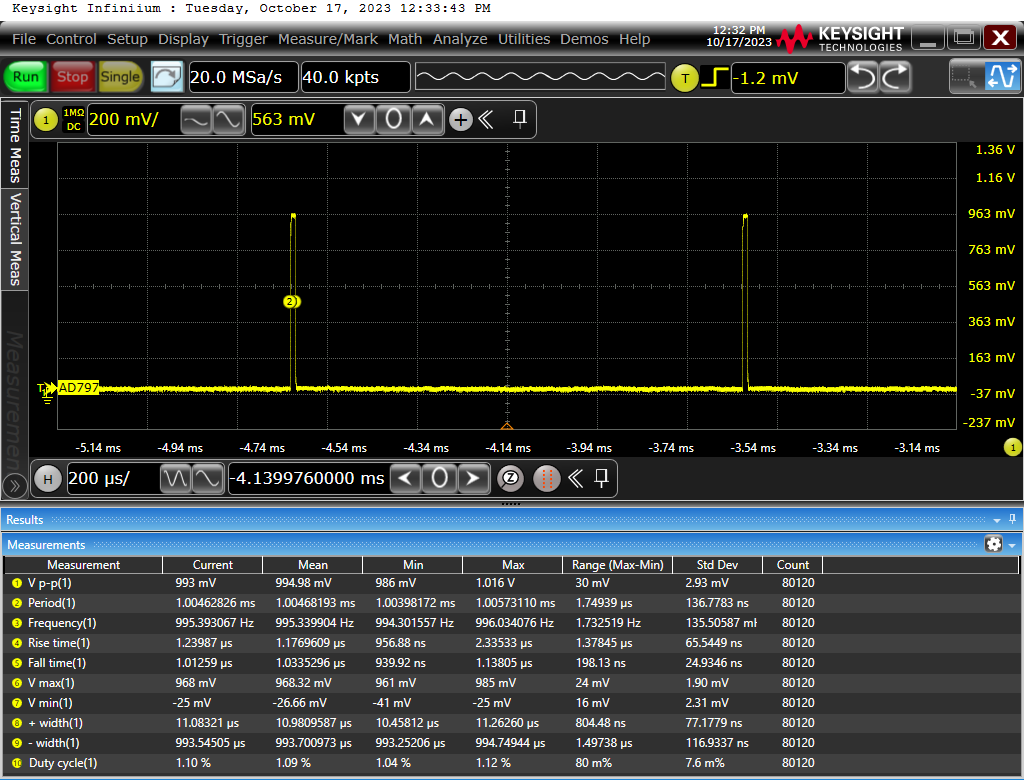

Supplement: Supplementary file 1 [file sensors-24-02883-s001.zip › Output waveform/10mA/0r01 0r1333.png]

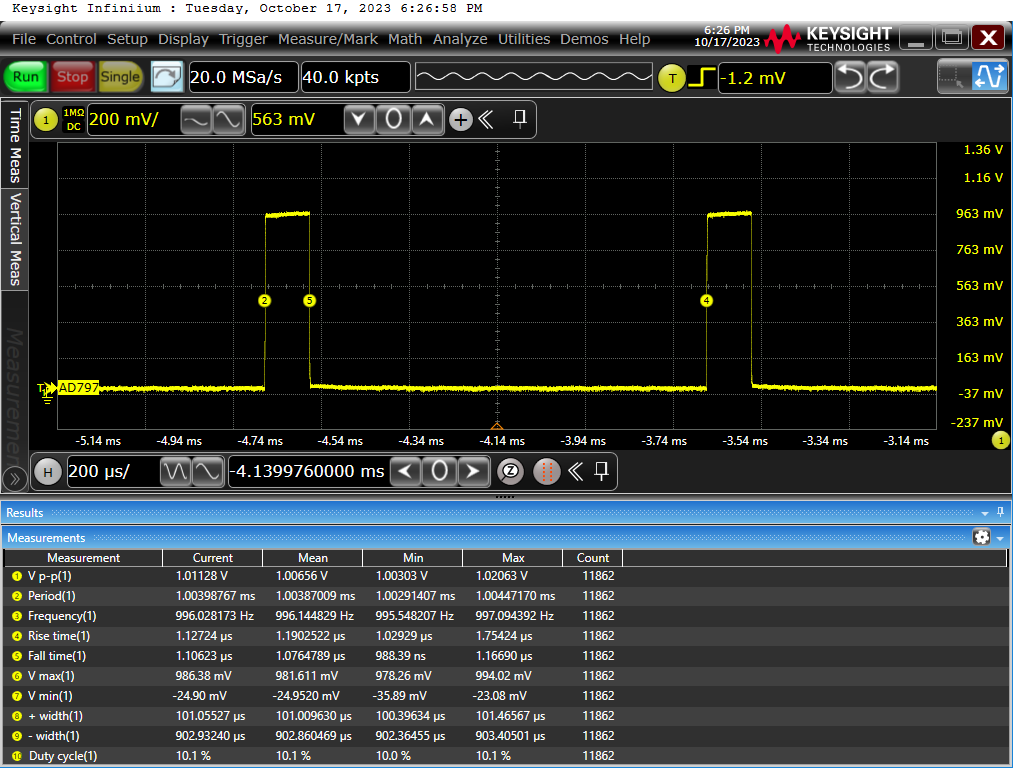

Supplement: Supplementary file 1 [file sensors-24-02883-s001.zip › Output waveform/10mA/0r10 0r627.png]

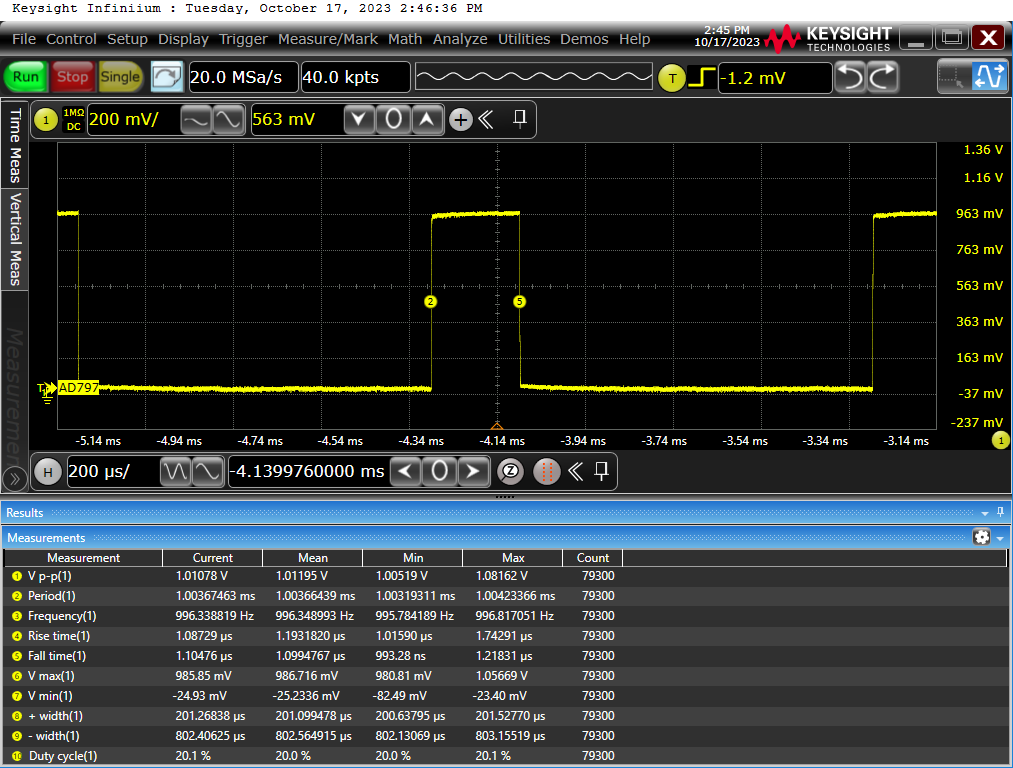

Supplement: Supplementary file 1 [file sensors-24-02883-s001.zip › Output waveform/10mA/0r20 1r08.png]

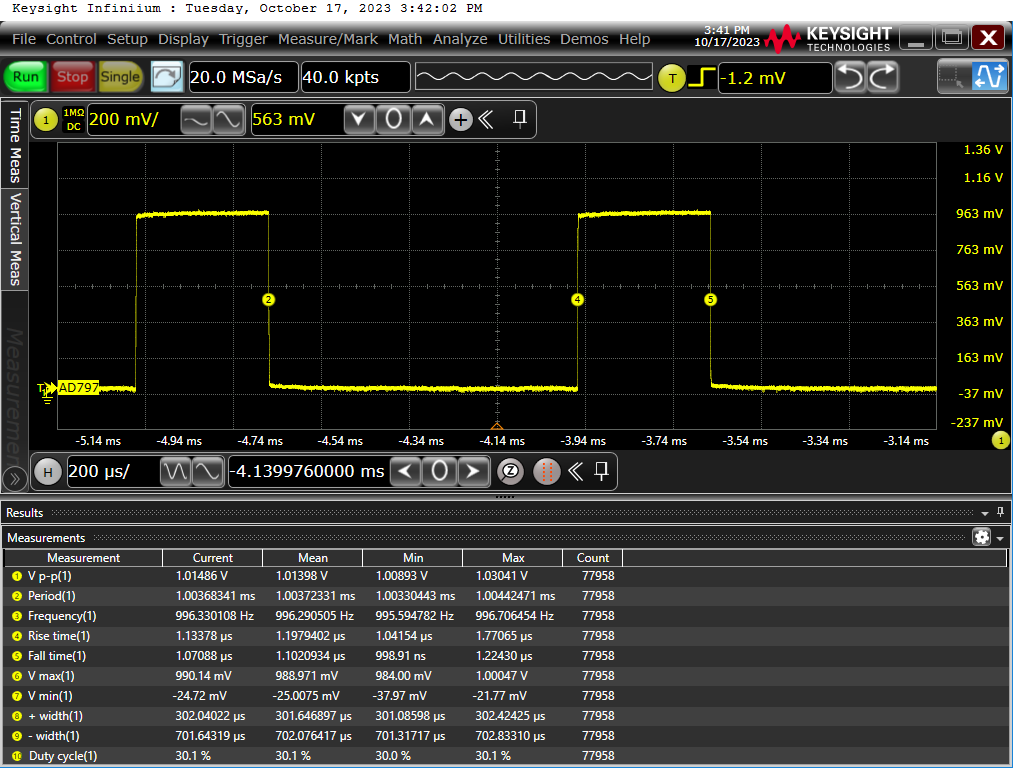

Supplement: Supplementary file 1 [file sensors-24-02883-s001.zip › Output waveform/10mA/0r30 1r506.png]

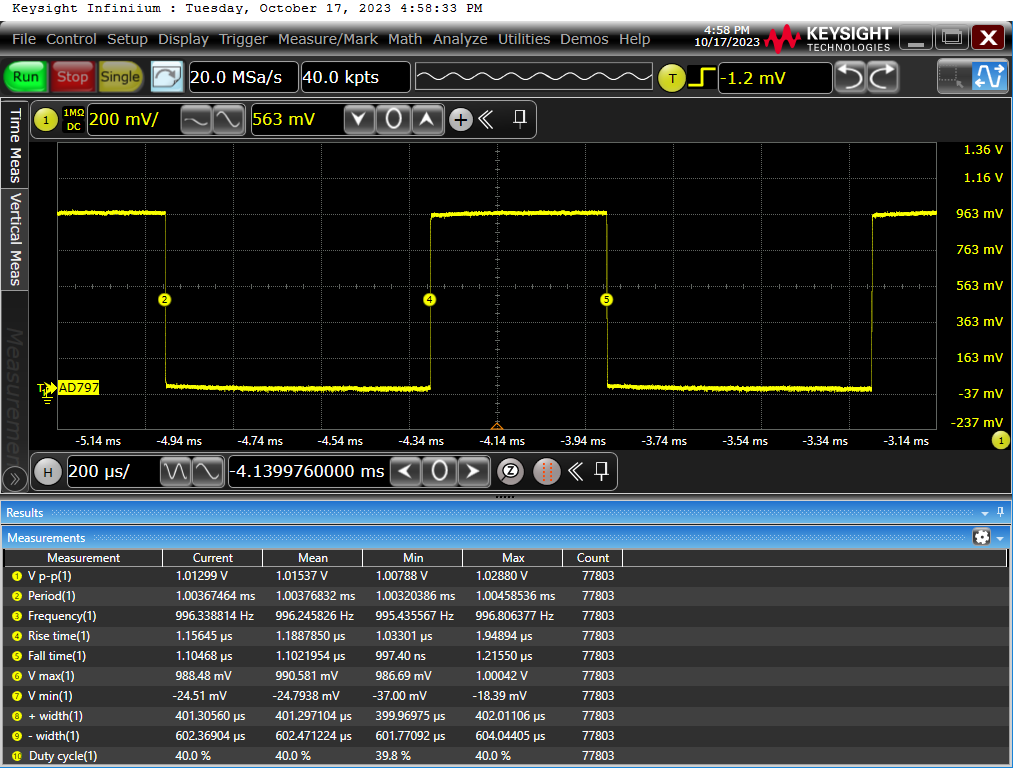

Supplement: Supplementary file 1 [file sensors-24-02883-s001.zip › Output waveform/10mA/0r40 1r915.png]

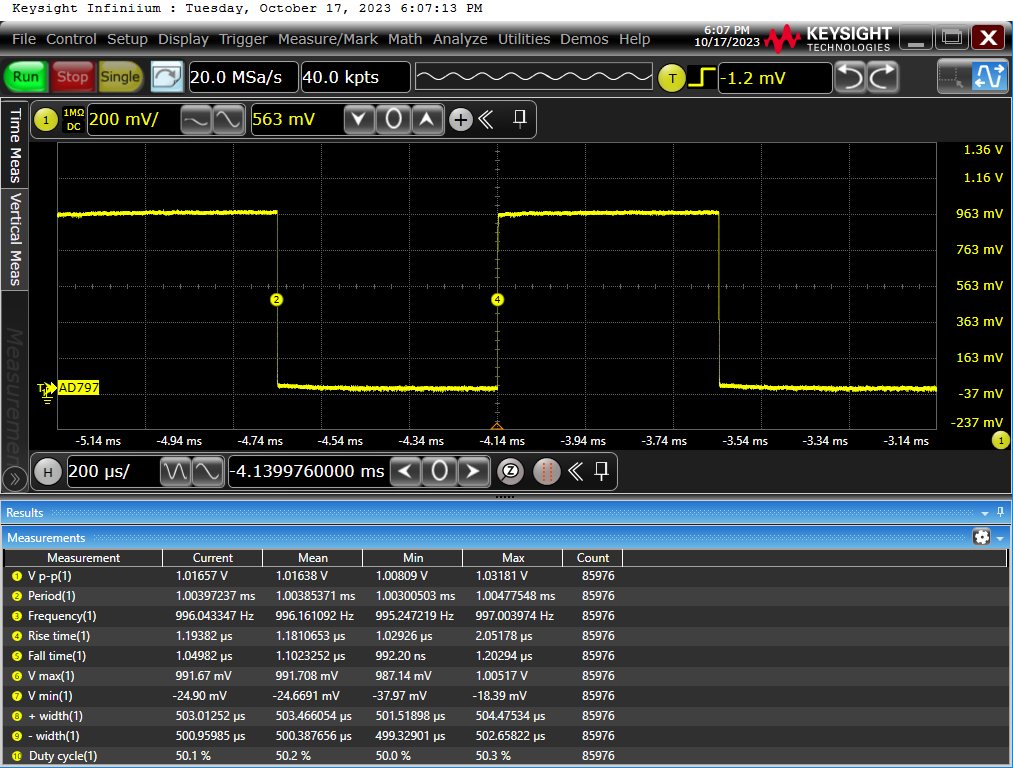

Supplement: Supplementary file 1 [file sensors-24-02883-s001.zip › Output waveform/10mA/0r50 2r326.png]

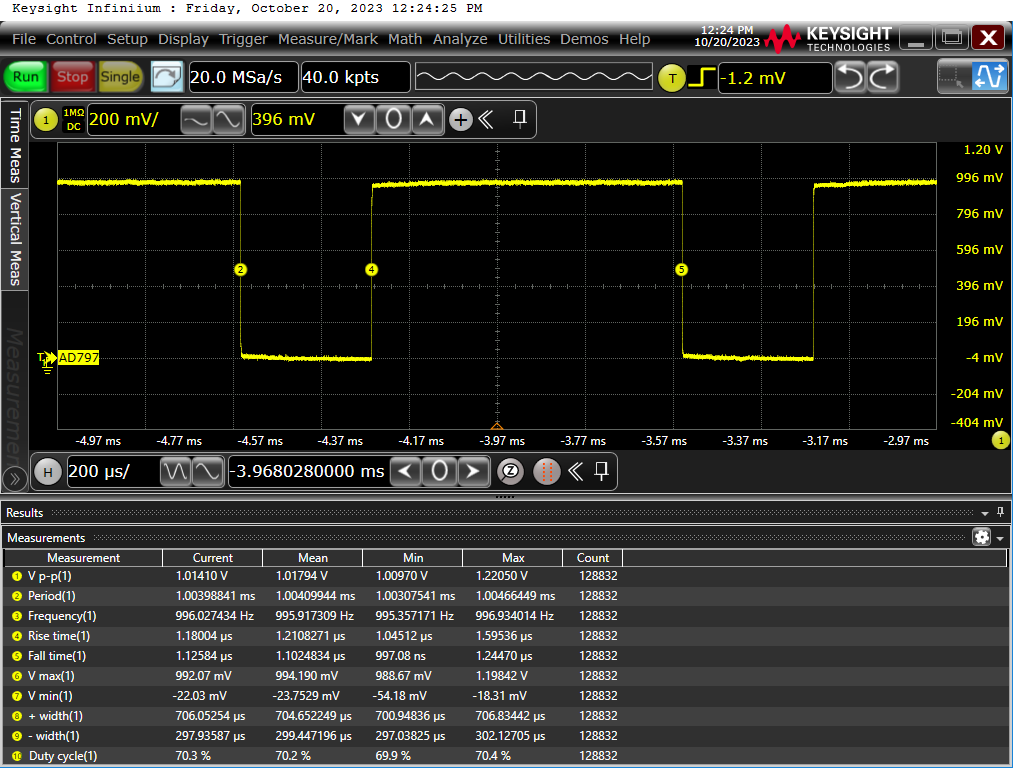

Supplement: Supplementary file 1 [file sensors-24-02883-s001.zip › Output waveform/10mA/0r70 3r026.png]

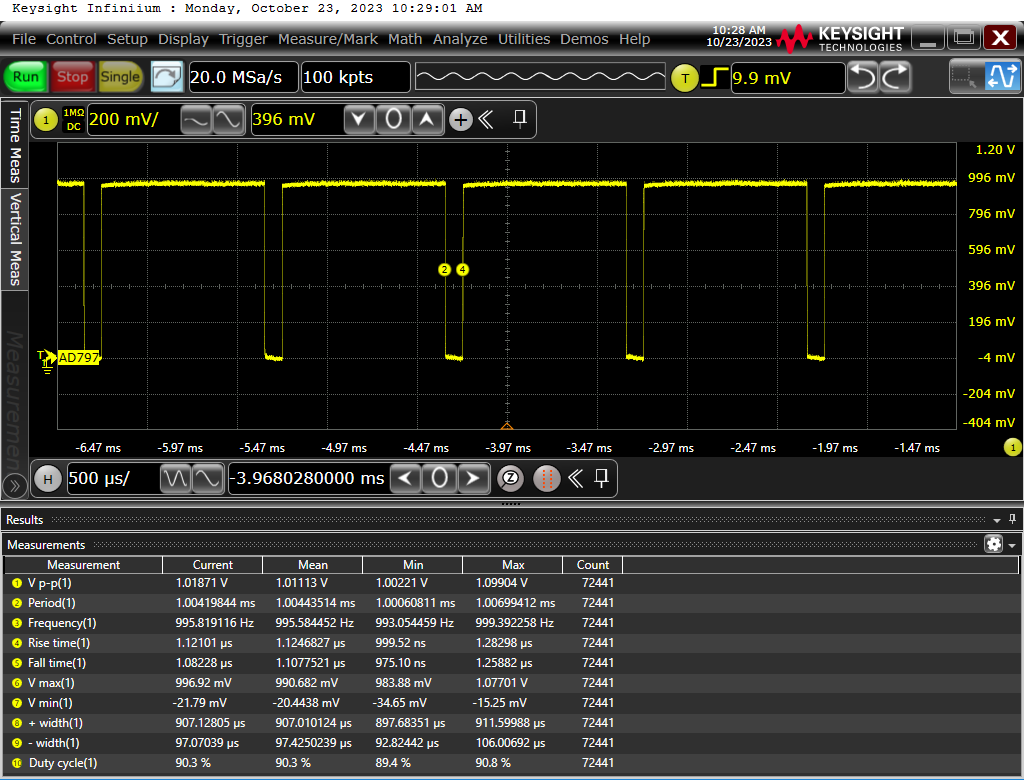

Supplement: Supplementary file 1 [file sensors-24-02883-s001.zip › Output waveform/10mA/0r90 3r853.png]

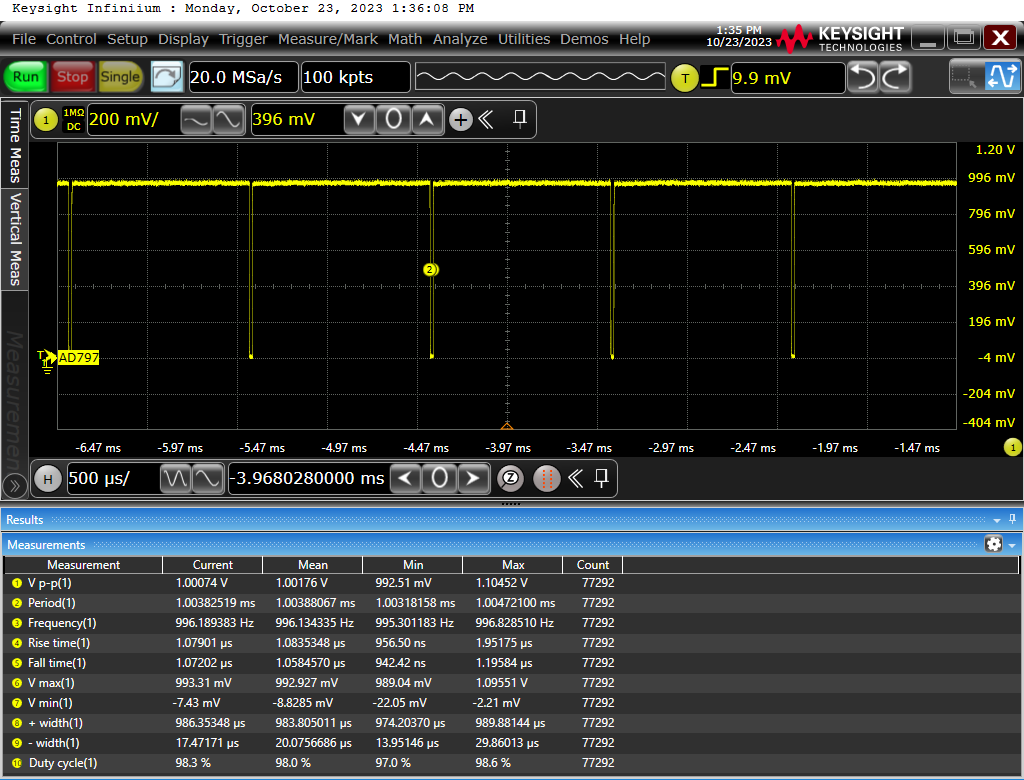

Supplement: Supplementary file 1 [file sensors-24-02883-s001.zip › Output waveform/10mA/0r98 4r188.png]

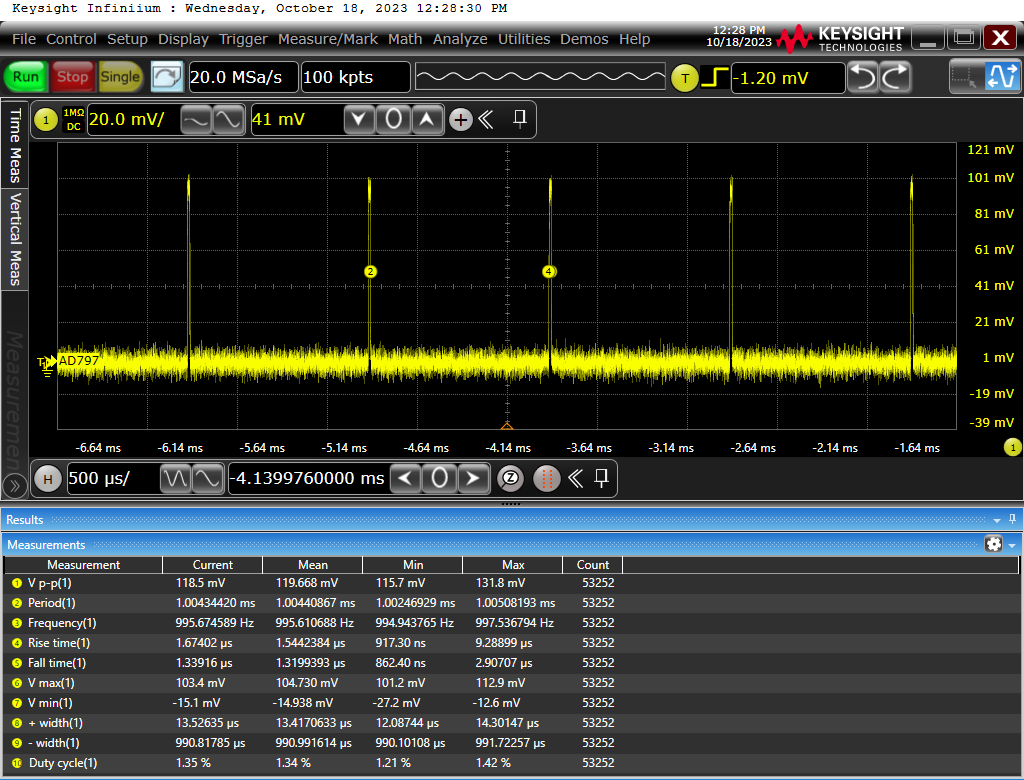

Supplement: Supplementary file 1 [file sensors-24-02883-s001.zip › Output waveform/1mA/0r01 0r02564.png]

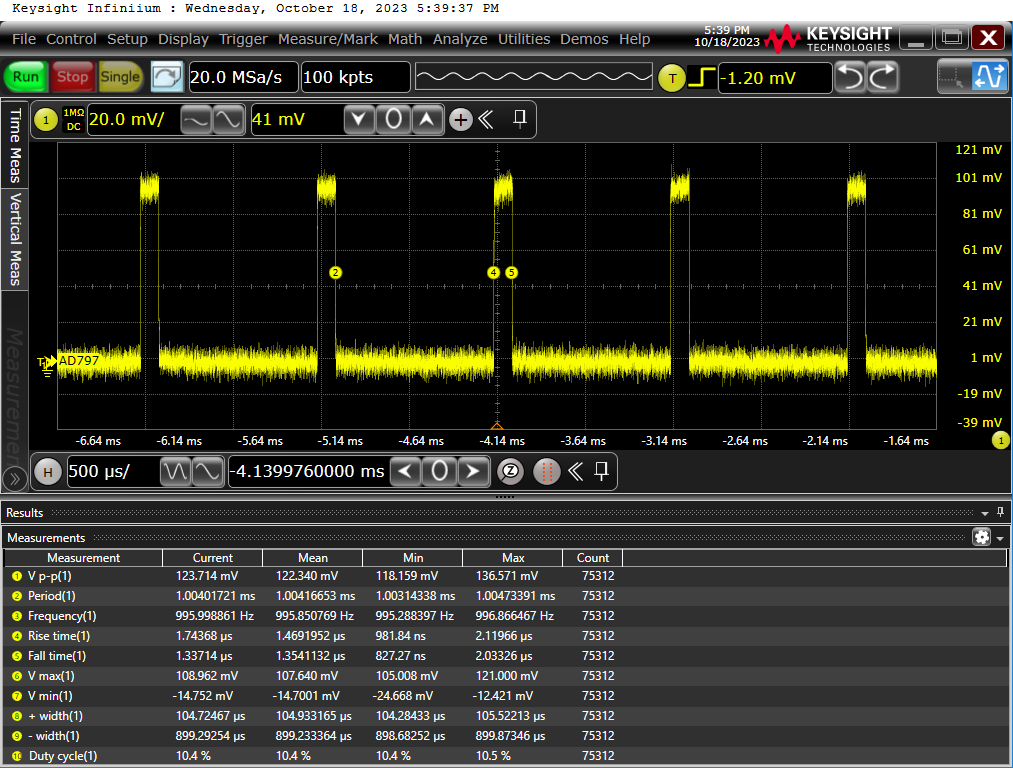

Supplement: Supplementary file 1 [file sensors-24-02883-s001.zip › Output waveform/1mA/0r10 0r15239.png]

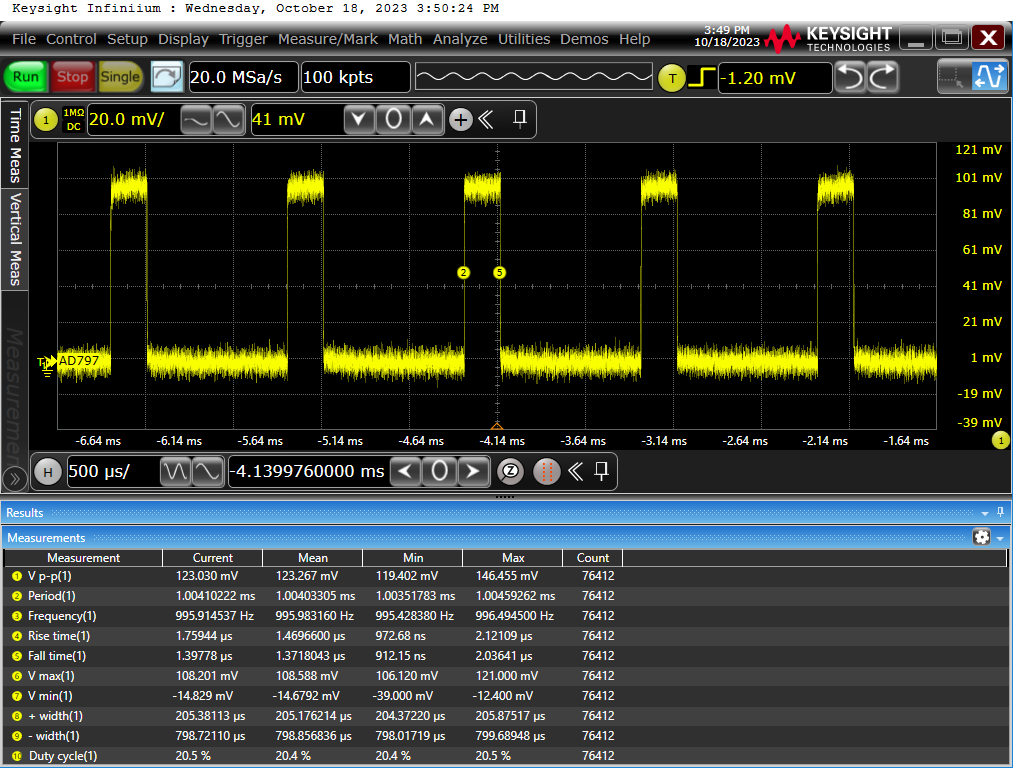

Supplement: Supplementary file 1 [file sensors-24-02883-s001.zip › Output waveform/1mA/0r20 0r28447.png]

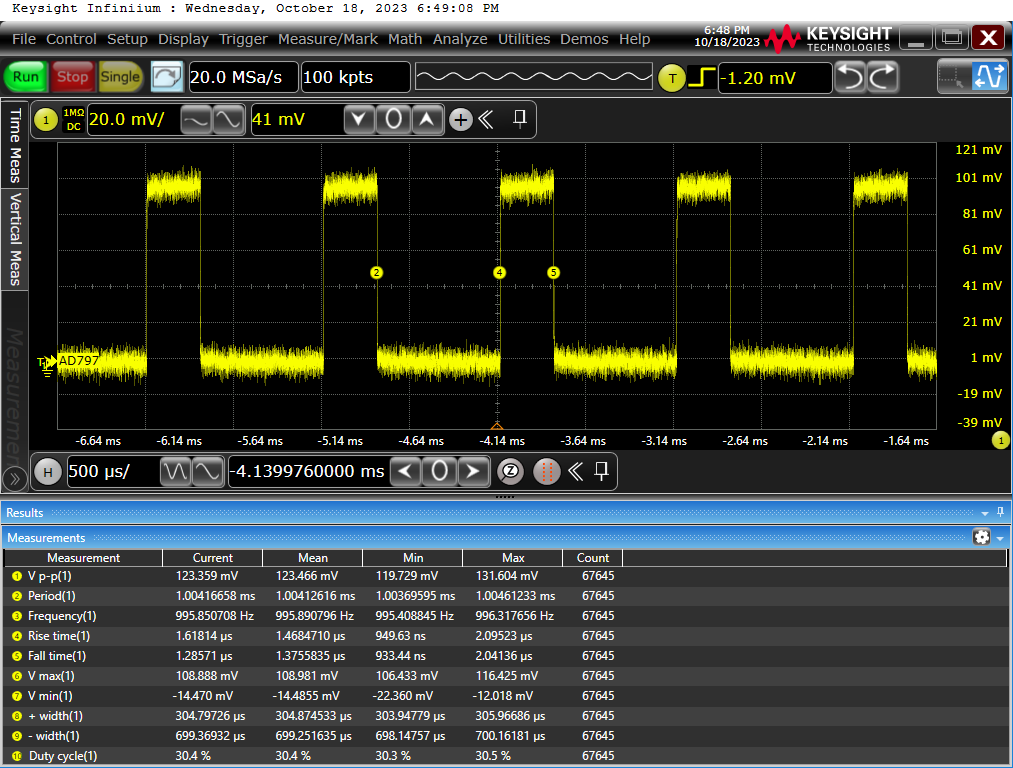

Supplement: Supplementary file 1 [file sensors-24-02883-s001.zip › Output waveform/1mA/0r30 0r4138.png]

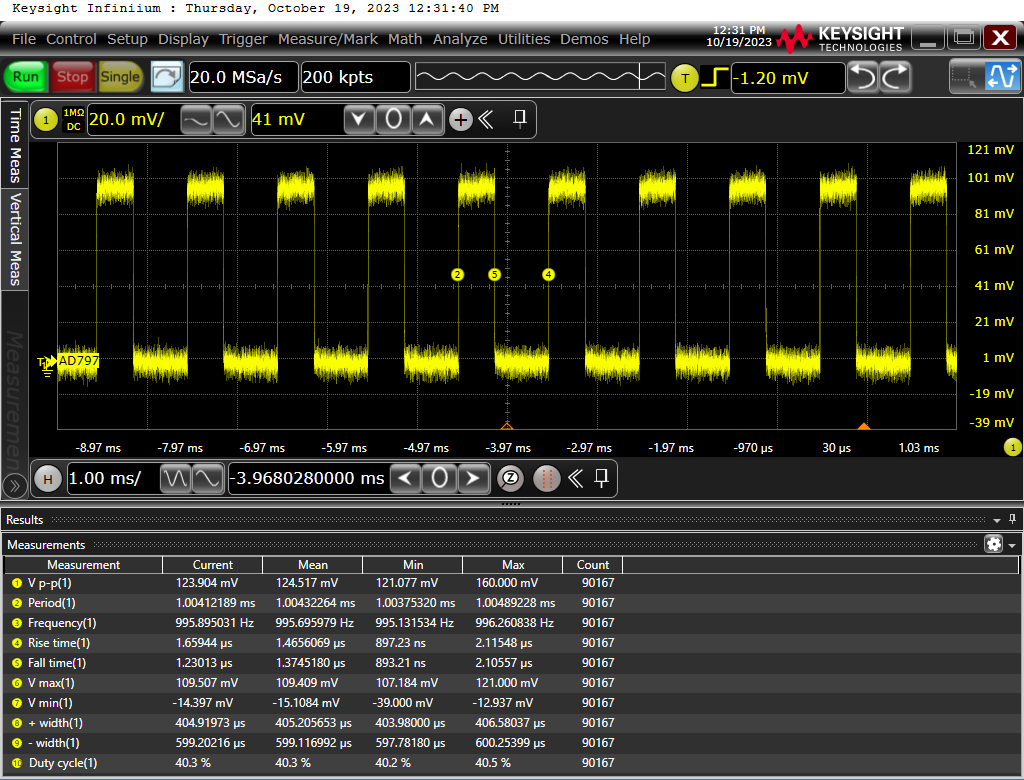

Supplement: Supplementary file 1 [file sensors-24-02883-s001.zip › Output waveform/1mA/0r40 0r5445.png]

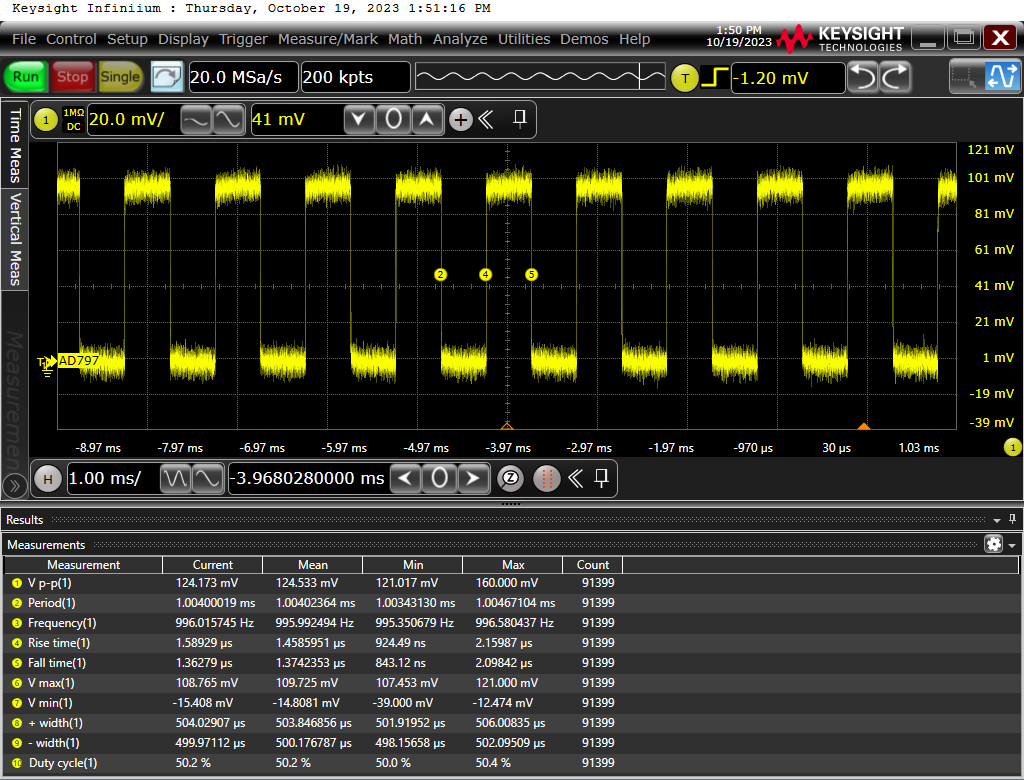

Supplement: Supplementary file 1 [file sensors-24-02883-s001.zip › Output waveform/1mA/0r50 0r6766.png]

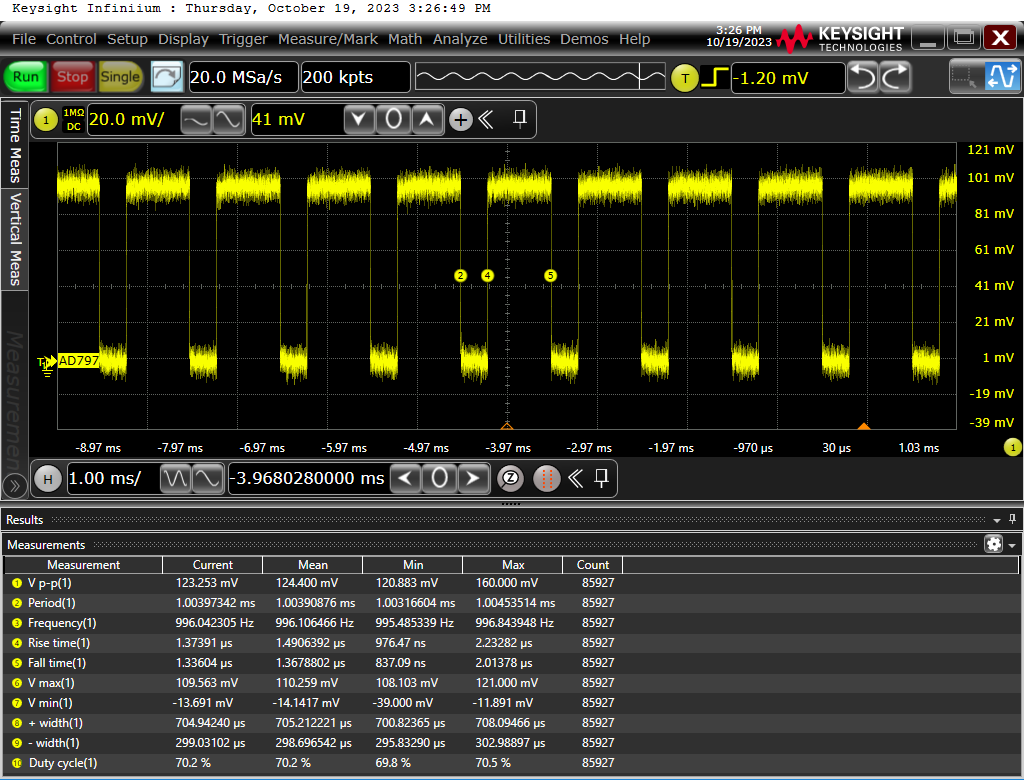

Supplement: Supplementary file 1 [file sensors-24-02883-s001.zip › Output waveform/1mA/0r70 0r955.png]

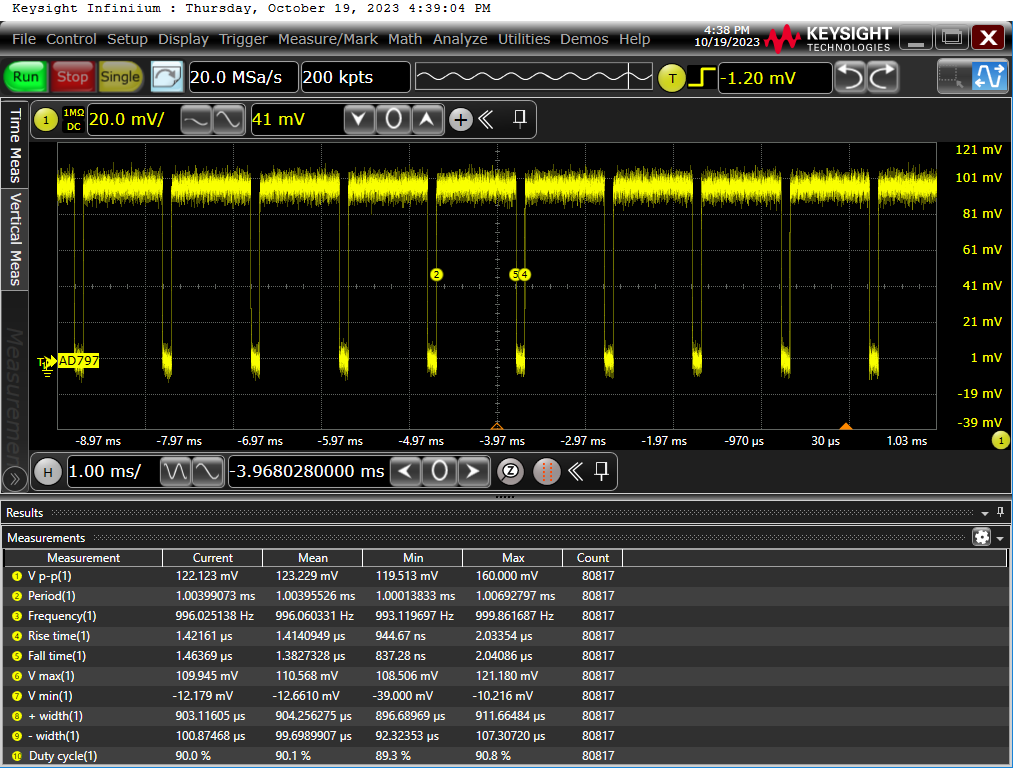

Supplement: Supplementary file 1 [file sensors-24-02883-s001.zip › Output waveform/1mA/0r90 1r234.png]

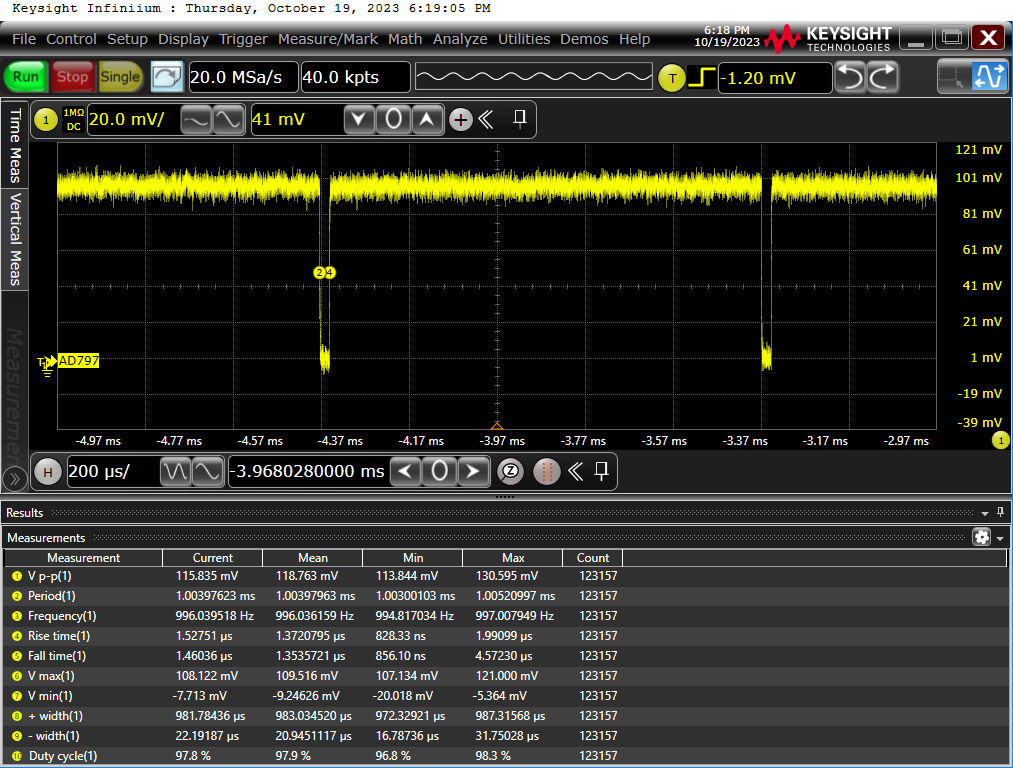

Supplement: Supplementary file 1 [file sensors-24-02883-s001.zip › Output waveform/1mA/0r98 1r354.png]
